# Adding Virtual Reality Mindful Exposure Therapy to a Cancer Center’s Tobacco Treatment Offerings: Feasibility and Acceptability Single-Group Pilot Study

**DOI:** 10.2196/54817

**Published:** 2024-07-23

**Authors:** Riley Walton Jackson, Ann Cao-Nasalga, Amy Chieng, Amy Pirkl, Annemarie D Jagielo, Cindy Xu, Emilio Goldenhersch, Nicolas Rosencovich, Cristian Waitman, Judith J Prochaska

**Affiliations:** 1 Department of Medicine Stanford University Stanford, CA United States; 2 Health Education, Engagement and Promotion Stanford Health Care Stanford, CA United States; 3 Stanford Prevention Research Center Department of Medicine Stanford University Palo Alto, CA United States; 4 PGSP-Stanford PsyD Consortium Palo Alto University Palo Alto, CA United States; 5 Laboratorio de Investigación en Neurociencia y Ciencias Sociales Universidad de Flores Buenos Aires Argentina; 6 Escuela de Ingeniería Biomédica Universidad Nacional de Córdoba Córdoba Argentina; 7 Universidad Siglo XXI Córdoba Argentina

**Keywords:** tobacco cessation, virtual reality, exposure therapy, cancer care, mobile phone

## Abstract

**Background:**

Smoking contributes to 1 in 3 cancer deaths. At the Stanford Cancer Center, tobacco cessation medication management and counseling are provided as a covered benefit. Patients charted as using tobacco are contacted by a tobacco treatment specialist and offered cessation services. As a novel addition, this study examined the acceptability of a virtual reality (VR) mindful exposure therapy app for quitting smoking called MindCotine.

**Objective:**

The objective of this study was to determine the feasibility and acceptability of offering 6 weeks of MindCotine treatment as a part of Stanford’s Tobacco Treatment Services for patients seen for cancer care.

**Methods:**

As part of a single-group pilot study, the MindCotine VR program was offered to English- or Spanish-speaking patients interested in quitting smoking. Given the visual interface, epilepsy was a medical exclusion. Viewed from a smartphone with an attachable VR headset, MindCotine provides a digital environment with audiovisual content guiding mindfulness exercises (eg, breathing techniques, body awareness, and thought recognition), text-based coaching, and cognitive behavioral therapy-based self-reflections for quitting smoking. Interested patients providing informed consent were mailed a MindCotine headset and asked to use the app for 10+ minutes a day. At the end of 6 weeks, participants completed a feedback survey.

**Results:**

Of the 357 patients reached by the tobacco treatment specialist, 62 (17.3%) were ineligible, 190 (53.2%) were not interested in tobacco treatment services, and 78 (21.8%) preferred other tobacco treatment services. Among the 105 eligible and interested in assistance with quitting, 27 (25.7%) were interested in MindCotine, of whom 20 completed the informed consent, 9 used the program, and 8 completed their end-of-treatment survey. Participants using MindCotine completed, on average, 13 (SD 20.2) program activities, 19 (SD 26) journal records, and 11 (SD 12.3) coaching engagements. Of the 9 participants who used MindCotine, 4 (44%) reported some dizziness with app use that resolved and 7 (78%) would recommend MindCotine to a friend. In total, 2 participants quit tobacco (22.2% reporting, 10% overall), 2 others reduced their smoking by 50% or more, and 2 quit for 24 hours and then relapsed.

**Conclusions:**

In a feasibility and acceptability pilot study of a novel VR tobacco treatment app offered to patients at a cancer center, 4 of 9 (44%) reporting and 4 of 20 (20%) overall substantially reduced or quit using tobacco after 6 weeks and most would recommend the app to others. Further testing on a larger sample is warranted.

**Trial Registration:**

ClinicalTrials.gov NCT05220254; https://clinicaltrials.gov/study/NCT05220254

## Introduction

Tobacco use, and in particular cigarette smoking, has taken a devastating toll on public health. While tobacco smoking has declined significantly over the past several decades, it remains the leading cause of preventable disease, disability, and death in the United States [1] and worldwide. Smoking contributes to 1 in 3 cancer deaths [2,3], and more than half of all people who smoke long term will die from a tobacco-related disease, losing 10 years of life on average [2].

Quitting smoking proves to be a difficult task, even when individuals have a strong desire to stop. In 2015, nearly 7 in 10 US adults (22.7 million) who smoked wanted to quit [4]; and in 2018, 55.1% of adults who smoked (21.5 million) reported quitting for at least 1 day in the prior year [5]. Of the 7.5% (2.9 million) who had quit at 6 months in 2018 [5], a majority (>50%) are anticipated to have relapsed by 1 year [6].

Nicotine’s effects are transient, and within an hour of having a cigarette, withdrawal symptoms can begin. These include irritability, anxiety, depressed mood, and difficulty concentrating [2]. There is also a behavioral component to tobacco use disorder [2,7]. The act of smoking becomes associated with the pleasurable effects of nicotine and functions as a coping mechanism when individuals feel negative emotions [2,7,8]. Furthermore, environmental and situational factors, such as being around friends who smoke or watching television, can become linked with nicotine and act as triggers or cues for usage [2,9]. Thus, effective treatments often combine multiple strategies to target both the behavioral and physiological aspects of nicotine dependence [2,7,8].

Studies of smoking and different forms of cancer indicate that between 35% and 72% of patients who smoke continue to smoke after cancer diagnosis and treatment [3,10]. Continuing to smoke during treatment can decrease the effectiveness of radiotherapy, wound healing efficiency, and survival time [3,10]. Smoking can also increase the risk of treatment side effects and complications, cancer recurrence, and second primary malignancies [3,10]. Quitting smoking can improve treatment outcomes and survival [3]. Cancer screening, diagnosis, and hospitalization can motivate and support a quit attempt, making it a critical time for engagement with cessation resources and treatment [1,3,10].

Traditional treatment strategies for quitting smoking include behavioral counseling (individual or group) and cessation medications [1,2,7], such as nicotine replacement therapy (NRT). These evidence-based treatments increase quit rates when used, and clinical practice guidelines recommend using them in combination [7,8]. However, not all individuals are receptive to or able to take cessation medications [11]. Behavioral counseling might not be available or preferable to patients due to travel, time, and cost constraints, in addition to concerns about privacy or feelings of shame (eg, in group counseling) [2,12].

To address these challenges, additional delivery methods for smoking cessation interventions have been developed. As of 2021, 85% of US adults own a smartphone, and there is immense potential for reach, accessibility, flexibility, and cost-effectiveness of mobile health interventions [2,13,14]. Mobile health interventions can ideally be combined with pharmacotherapy and counseling, or they may be used as standalone therapies for patients who are not interested in or candidates for cessation medications and traditional behavioral counseling. There is evidence for the efficacy of Internet, Quitline, and text-based cessation resources, as well as preliminary evidence for mobile apps [1,2,12,13]. Evidence for the efficacy of technology-based interventions in smoking cessation is limited yet promising [13,15].

An emerging field for the treatment of mental health disorders, including substance use disorders, is virtual reality (VR). VR generally refers to an interactive, computer-generated experience that consists of a simulated, immersive environment [12,16,17]. VR can be used to create digital environments that elicit challenging sensations and emotions, which can be used to create opportunities for learning and practicing skills to manage them [9,12,16,17]. VR immersion and interactivity can vary with different types of headsets (smartphone app-based, stand-alone, and tethered) and apply different theoretical approaches (eg, cognitive behavioral, gamified behavioral, and cue exposure) with varying outcomes [18]. The most established evidence for the use of VR in treatment is exposure-based treatment for anxiety disorders [16,17]. There is a small but growing body of evidence indicating that VR is effective in inducing cravings for cigarettes, thus allowing for the opportunity to practice virtually presented coping strategies [9,12,16-18].

A randomized controlled pilot study on the efficacy of a smartphone app-based VR mindful exposure therapy (VR-MET) smoking cessation program developed by MindCotine was conducted in 2018. VR-MET incorporated mindfulness training and cue exposure as part of the VR intervention. While VR sessions typically occur in a laboratory or hospital-based setting with supervising staff, MindCotine VR sessions are able to be self-administered and delivered remotely [9,12]. With a headset that connects to a smartphone, MindCotine creates an immersive environment in a cost-effective and scalable manner [12]. This contrasts with other forms of VR that are less immersive (use a computer monitor only) or highly expensive, such as head-mounted systems with separate displays for each eye [9,17]. Preliminary evidence shows that mindfulness interventions can be efficacious for smoking cessation in traditional, in-person, and technology-based formats [12,15]. Additionally, evidence supports that cognitive behavioral therapy (CBT) is effective as a smoking cessation treatment when delivered in a traditional format [13,15]. CBT focuses on identifying and changing maladaptive thoughts and behavioral patterns to alleviate distress. The app uses a series of CBT reflective questions and journaling for participants to evaluate changes in their relationship with smoking and offers text-based coaching. In this randomized controlled study, a sample of 120 individuals who smoked at least 5 cigarettes a day living in Buenos Aires, Argentina, was recruited via Facebook and television advertisements, with 93% (56/60) of the sample completing the full 21-day program [12]. Individuals in the control condition received a self-help manual on smoking cessation created by the Argentine Ministry of Health. After the intervention, the treatment group participants reported a significant decrease in cigarettes per day, lower cravings, increased readiness to quit, and higher abstinence rates compared to the control group [12]. Overall, the MindCotine intervention was found to be feasible and acceptable, with preliminary evidence supporting smoking cessation in a nonclinical community sample. The use of the VR-MET app in a clinical sample and among people with lower levels of smoking remains unexplored. With a focus on integrated cancer care, we aimed to test VR-MET as a novel tobacco cessation treatment in addition to our clinic’s offerings.

In 2018, the Stanford Cancer Institute integrated tobacco treatment services within cancer care as part of the Cancer Center Cessation Initiative [3]. Collaboration with 3 pilot clinics began in 2019 (head and neck, thoracic, and gastrointestinal), and by 2020, the Stanford Tobacco Treatment Service had successfully extended into more than 20 clinics at the cancer center [3]. The service continues to explore expansion opportunities to improve patient reach and engagement during cancer care, including collaborating with MindCo Health to explore VR smoking cessation with this pilot study.

The aim of this study was to determine the feasibility and acceptability of a VR tobacco cessation treatment among patients seen for cancer care who smoke. If feasible and acceptable, the intervention could expand the treatment options available to patients, potentially improve cessation rates, and help improve cancer care outcomes. A secondary objective was to study the efficacy of the intervention in a clinical sample. Furthermore, the COVID-19 pandemic has altered traditional health care delivery. Many patients are immunocompromised due to their anticancer treatment or the disease itself, and there has been an increase in the use of telehealth services for cancer care to avoid unnecessary exposure to COVID-19 [19]. Thus, the implementation of a remote delivery smoking cessation treatment may be particularly beneficial for patients undergoing cancer treatment.

This pilot differs from the initial efficacy trial by sample. The initial efficacy trial studied adults with a high level of daily smoking residing in Buenos Aires, Argentina, while this study focused on adult patients of the Stanford Cancer Center who smoked daily or nondaily [12]. Patients with cancer face unique barriers as participants in research studies, including lasting side effects of treatment and coordinating many different care providers [20]. Participating in a research study may seem overwhelming and difficult to manage during cancer treatment. However, the Tobacco Treatment Service at Stanford has previously (in 2019) been able to reach 74% (273/368) of patients identified as using tobacco and engage 33% (90/273) of the patients reached in cessation treatment services [3]. Treating smoking can greatly improve cancer care outcomes [10].

## Methods

### Study Design

This was a single-group pilot study designed for a maximum of 20 participants, assessing outcomes after 6 weeks of use. The primary objective was to determine the feasibility and acceptability of this VR intervention. Feasibility and acceptability metrics were at least (1) 20% of participants interested in using the app; (2) 75% of interested patients onboarded and using the app; and (3) 70% of participants recommending the app to others. The secondary objective was to capture efficacy by evaluating quit attempts, smoking reduction, and abstinence. Our goals were at least 50% of participants reducing tobacco use and 10% of them quitting. The metrics and tobacco usage were assessed via a survey at enrollment and at the end of treatment.

### Ethical Considerations

Study approvals were obtained from the Stanford University Institutional Review Board on May 18, 2021 (protocol 60253), and the Stanford Cancer Scientific Review Committee on September 19, 2021 (PS0020). There was no compensation for participation in the study. The study was offered as an option as part of the Stanford Tobacco Treatment Service. All study materials were provided to participants at no cost, and the VR kits did not need to be returned.

The study had a waiver of documentation to allow for telephone consent to screen and an eligibility questionnaire. The eligibility questionnaire also functioned as the baseline questionnaire for those eligible for the study. Informed consent procedures used the Adobe Sign platform and were compliant with HIPAA (Health Insurance Portability and Accountability Act) and 21CFR11. Study staff was available via the phone to navigate consent. Each potential participant was able to take time to consider participation and could drop out at any time, knowing their participation was voluntary. No other tobacco treatment options were withheld. Materials were available in English and Spanish. Stanford Health Care Interpreter Services could be used during consent as needed.

Assessment data were collected and stored via REDCap (Research Electronic Data Capture), a HIPAA-compliant survey administration tool developed by Vanderbilt University. The MindCotine app was reviewed by Stanford’s Data Risk Assessment team prior to the start of recruitment. The study also underwent annual reviews by the Stanford Cancer Center Data Safety Monitoring Committee.

The Stanford clinical team handled the outreach portion and informed the research team about which of the patients would like to participate in the study. The Stanford research team managed the mailing of kits, the end of treatment, and data analysis. MindCotine had access to the app user data, which was provided to the Stanford research team through secure email. The Stanford research team provided a summary of outcomes (no PHI) to MindCotine.

### Recruitment

Study recruitment occurred between November 16, 2021, and August 18, 2022. Patients seen for cancer care at the Stanford Cancer Center, who were charted as using tobacco products in the electronic health record, were included in a weekly report that the tobacco treatment specialist used to reach out to patients offering tobacco treatment services. Patients were offered a variety of treatment options, including individual counseling, group counseling, cessation medications (eg, NRT, varenicline, and bupropion), and the MindCotine VR intervention. The MindCotine intervention was described to patients as a “mobile-based virtual reality program for quitting smoking,” and they were told that “the program teaches skills for quitting smoking and is very visual. The treatment can be done at home from your phone.”

Patients who expressed interest in MindCotine completed a phone screen to determine eligibility. Inclusion criteria were smoking daily or nondaily (at least 9 cigarettes per week), fluency in English or Spanish, owning a compatible smartphone, being open to assistance with quitting smoking, and willing to use the MindCotine app for at least 10 minutes daily for 6 weeks and to complete survey assessments. Exclusion criteria were a history of seizures or an epilepsy diagnosis.

### Study Procedures

Patients deemed eligible went through an informed consent process and were sent a password protected link to read and e-sign the informed consent form. Once consent was completed, a MindCotine VR kit was mailed to participants, which included the cardboard or plastic headset and an activation code to gain full access to the app for a year at no cost. The app shared suggestions, such as sitting comfortably and using headphones, before the VR experience. Users could also schedule meetings with the MindCotine app coaching team, where further explanation on regulating the focus with the VR headset could be provided. Instructions were delivered by phone or video call. At the end of the 6 weeks, participants were contacted by a research team member to complete an end-of-treatment assessment.

### Intervention

This study used the MindCotine smartphone app for the VR smoking cessation intervention. The MindCotine intervention consists of audiovisual media with VR and non-VR content drawing on mindfulness-based techniques and CBT strategies for addressing smoking cravings [12]. Treatment components within the MindCotine app are summarized in Table 1. VR sessions focus on the act of smoking and cravings, stress at work, and bodily sensations. The VR intervention included 7 VR-MET sessions, each lasting 10 minutes. These experiences consist of a combination of animated environments at the beginning and end of each session lasting 4 minutes total to induce meditative states, and 6 minutes of cue-exposure therapy filmed with a 360 camera. The reason behind this integrated approach is based on studies showing the use of VR in natural environments to create relaxation and VR with real people to simulate familiar situations to elicit cravings and bodily sensations [21,22]. Non-VR sessions focus on formal mindfulness-based exercises such as deep breathing, body scans, and recognizing thoughts, emotions, and the impulse to smoke.

**Table 1:** MindCotine App Components for Treating Tobacco Use.

App component	Description of components	Virtual reality
Program activity	Virtual Reality—Mindful Exposure Therapy exercises and guided mindfulness-based 2-dimensional video, audio, and reflection exercises	Yes
Journal record	Cognitive behavioral therapy–based self-reflections. Includes tracking cigarette use per day	No
Coaching engagement	Communicating with a coach through the MindCotine mobile app	No
Lifesaver use	Brief informational intervention via chatbot	No
Input activity	Inputting an emotion experienced	No
Commitment activity	Setting a quit date	No

Participants were encouraged to engage with MindCotine for 6 weeks, with a different concept of focus each week (Table 2). A daily session of MindCotine varied between self-reflecting CBT questions, mindfulness audios or videos, and VR experiences, including daily journaling of cigarette consumption. The time varied between 1 and 10 minutes of usage. Due to the nature of VR-MET, every VR experience was embedded as part of the 6-week intervention to address the concepts (Table 2) in situations that were familiar to the patient and could train their emotional and practical coping skills in the comfort of their home while identifying the associated sensations elicited by the specific cues. All VR experiences were immersive but not interactive in order to reduce potential dizziness.

**Table 2:** Overview of 10-minute weekly concepts in the 6-week MindCotine intervention [19].

Week	Concept	Description
1	Self-awareness	Breathing techniques, the act of smoking, reasons and motivations to start the program
2	Sensations	Focusing on bodily sensations in relation to smoking and triggers
3	Emotions	Managing cravings born out of stress and feelings related to familiar situations
4	Cognition	Exploring cognitive distortions, beliefs, and activities led by curiosity and reflections
5	Readiness	Practicing empathy and past victories to determine future actions
6	Preparation	Setting new structures that can stimulate resilience and behavior activation tasks

### Statistical Analysis and Outcome Goals

Descriptive statistics were used to summarize tobacco use characteristics, changes in tobacco use, MindCotine app usage, and feedback on the MindCotine app. Feasibility (primary objective) was defined as (1) at least 20% of patients open to assistance with quitting reporting interest in using the MindCotine app, (2) 75% of the interested patients being fully onboarded and using the app, and (3) 70% of participants who complete end-of-treatment survey indicating that they would recommend the app to others. Efficacy (secondary objective) was defined as at least 50% of participants reducing their tobacco use and 10% or more quitting.

## Results

### Eligibility, Engagement, and Retention

**Figure 1:**
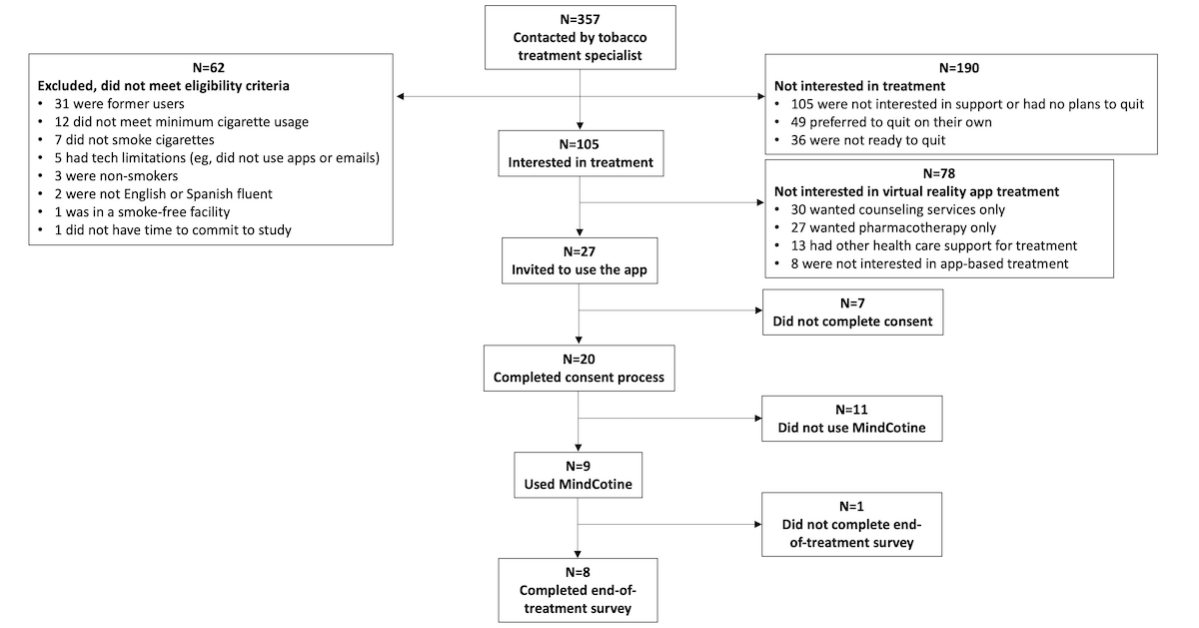
Participant flow diagram of before and after the MindCotine 6-week smoking cessation intervention among Stanford Cancer Center patients.

Of the 357 patients reached by the tobacco treatment specialist, 62 (17.3%) were ineligible, 190 (53.2%) were not interested in treatment for quitting tobacco, and 78 (21.8%) preferred other cessation treatment services (Figure 1). Of the 27 participants interested in MindCotine (25.7% of the 105 eligible and interested in quit assistance), 20 (74.1%) completed the consent process. Of the 20 participants, 9 (45%) used the MindCotine program, and 8 (40%) completed the end-of-treatment assessment.

### Baseline Sample Characteristics

Of the 20 participants who consented to participate, there were 11 women and 9 men; their mean age was 52 (SD 13, IQR 41-63) years; 15 identified as White, 2 as Asian, 1 as Black, 1 as multiracial, and 1 was unreported. All were non-Hispanic. At study start, 18 of 20 (90%) were smoking cigarettes daily and averaged 14.7 cigarettes (SD 7.2) per day (median 15, IQR 10-20). Among the 2 (10%) who reported smoking on some days, they averaged 25 cigarettes per week (SD 7, IQR 22-28). At baseline, 50% (10/20) were not using any other cessation support; 7 (35%) were using a combination of counseling services and cessation medication; 2 (10%) were only using cessation medications; and 1 (5%) was using counseling services only.

### MindCotine Use

**Figure 2:**
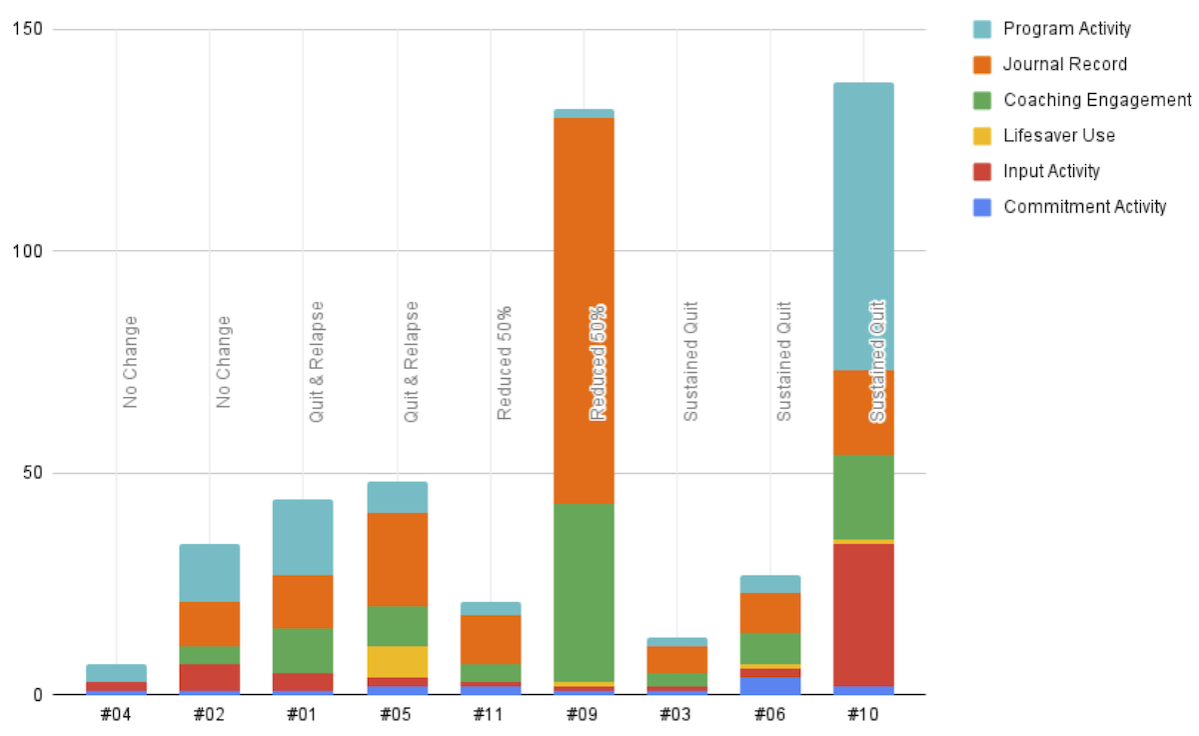
Participant usage of MindCotine virtual reality mindful exposure therapy program components during the study period (N=9).

Participants using MindCotine completed, on average, 13 (SD 20; median 4, IQR 3-13) program activities, 19 (SD 26; median 11, IQR 9-19) journal records, and 11 (SD 12; median 7, IQR 4-11) coaching engagements (Figure 2). In general, without limiting to the 6-week treatment period, active use of the MindCotine app ranged from 3 to 266 (mean 63, SD 80; median 48, IQR 15-56) days.

### Feasibility and Acceptability

Out of 3 conditions for the primary objective of feasibility and acceptability, two were met: (1) 25.7% (27/105) of those eligible and interested in help with quitting smoking were interested in MindCotine (surpassing goal of 20%); and (2) 78% (7/9) of those who used MindCotine would recommend the app to a friend (surpassing goal of 70%). Less than half (45%, 9/20) of the consented and interested patients, however, were fully onboarded and used the app (goal of 75% not met).

Of the 9 participants who used the MindCotine app, 8 completed the end-of-treatment survey and provided feedback about the program. Out of 8 participants, 7 would recommend MindCotine to a friend (88% of those reporting, 78% of those who used the app). Out of 8 participants, 5 (62.5%) provided positive comments about their experience with the app, noting that it was enjoyable, fun, and helpful for people who want to quit smoking. Additionally, there were several points recommended for improvement or consideration when using the program. For example, 3 of 8 (37.5%) participants described headset discomfort and issues of alignment between the headset and smartphone that negatively impacted their experience. A total of 2 (25%) participants reported having difficulty getting started with the program and required assistance from clinic staff or MindCotine staff to set up the program. Out of 8 completing the end-of-treatment survey, 3 (38%) reported mild to moderate VR-related symptoms (eg, headache, disorientation, dizziness, motion sickness, and nausea) with app use. One additional participant who withdrew mentioned experiencing dizziness and lightheadedness. In all cases, the removal of the headset or discontinuing use of the app resolved the symptoms as expected.

### Smoking Outcomes

One of the 2 conditions for the secondary objective of efficacy was met. Smoking outcomes were available from both the MindCotine app and the end-of-treatment survey. Of the 9 participants with smoking outcomes, 2 quit tobacco (22% reporting, 10% overall [2/20]; goal: 10%), 2 additional participants reduced their smoking by 50% or more (44.4% reporting, 20% overall [4/20]; goal: 50%), and 2 quit for 24 hours then relapsed. The mean change in cigarette use was –4.1 cigarettes per day (SD 8; IQR –8 to –1). Of the 8 completing their end-of-treatment survey, 5 (62%; 25% overall) were using cessation medications (eg, NRT, varenicline, and bupropion).

## Discussion

### Principal Results

Out of 3 conditions for the primary objective of feasibility and acceptability, two were met: (1) 25.7% (27/105) of those eligible and interested in help with quitting smoking were interested in MindCotine (surpassing goal of 20%); and (2) 78% (7/9) of those who used MindCotine would recommend the app to a friend (surpassing goal of 70%). One of the 2 conditions for the secondary objective of efficacy was met: 2 of the 9 participants with smoking outcomes quit tobacco (22% reporting, 10% overall [2/20]; goal: 10%). Outcomes for feasibility, acceptability, and efficacy show promise, but with less than half of the consented patients using the app, further testing of MindCotine in a larger sample is warranted.

In this study, we were successful in reaching 357 patients to offer cessation services. A majority (53.2%, 190/357) declined assistance with quitting smoking, and 62 (17.4%) were not eligible for the study. Worth highlighting is that the patients contacted about the study were proactively called by the program’s tobacco treatment specialist after being identified as using tobacco by the Stanford Cancer Center. Barriers to accessing conventional cessation treatments, such as cost, travel, and coordination, were removed [1,2,12]. Therefore, it is unsurprising that not being ready to quit or declining cessation treatment were among the most common reasons for not participating in the MindCotine pilot. A higher level of participation would be anticipated in settings reliant on patients calling in (ie, reactive rather than proactive outreach models) who ostensibly would present with a higher interest in quitting smoking. Those who opted for cessation support other than MindCotine instead chose a health care provider, counseling, or pharmacotherapy (19.6%, 70/357). In Stanford’s Tobacco Treatment Service, patients initially start with 1 treatment option (typically 1:1 counseling) and may include another option later, as suggested by their individual counselor. This likely explains why a large proportion of patients not interested in the study cited their engagement with another cessation treatment or resource as the reason. Uptake of the MindCotine app could improve over time and could be optimized if integrated within counseling sessions. Adding a brief motivational video could also aid in the adoption of MindCotine. Though quit attempts are significantly more likely to be successful when individuals use cessation pharmacotherapy and behavioral counseling [8,23,24], most US adults who smoke try to quit without support, and most quit attempts fail [1]. Further research is needed to understand the hesitancy to engage in support for quitting smoking [23].

### Study Limitations

The low completion rate of the intervention made it difficult to draw conclusions about the efficacy of the MindCotine VR treatment. The small sample had limited demographic diversity. The study sample was not large enough to explore the effects of other smoking cessation options with or without the VR-MET treatment, which is worth exploring in future studies, especially for patients who have exhausted current treatment options as monotherapies. The field would benefit from more studies of VR treatment comparisons [18]. Additionally, this study had a short follow-up time (follow-up occurred immediately after program completion), and information on tobacco usage at baseline and end of treatment was self-reported rather than biochemically verified.

### Study Strengths

This study offered VR-MET as one of multiple treatment options for smoking cessation in a clinical sample undergoing cancer care. The menu of options allowed patients to choose what may work best for them without concern of being limited to 1 type of treatment, demonstrating practical application and outcomes in a health care setting.

While the scope of this pilot study is limited, there was demonstrated interest among patients undergoing cancer care in trying VR-MET for smoking cessation. Including people who have cancer in research studies may be challenging given the coordination of care and lasting side effects from treatment, but it remains extremely important for health and treatment recovery. Also worth testing is VR for smoking cessation and relapse prevention in other clinical samples where treatment outcomes are acutely affected by tobacco use, such as in the perioperative setting.

### Comparison With Prior Work

This pilot of MindCotine differed from an earlier efficacy trial in terms of enrollment and onboarding. The first was that participants in the efficacy trial were invited to meet with the research team in-person for consent, onboarding, and distribution of the MindCotine headset kit [12]. Most (80%, 120/150) of those invited to use the app confirmed their participation and completed the consent process, compared to 74% (20 of 27) in our pilot [12]. In our pilot, all aspects of the study occurred remotely. The remote method may have contributed to the slightly lower proportion of patients who completed the consent process.

In terms of scale and costs, smartphone app-based VR treatments have these benefits over standalone and tethered VR sets. The MindCotine VR-MET was a smartphone app-based treatment, which allowed for freedom of wherever the user decided to engage in smoking cessation treatment, and shipping of the VR kit did not involve handling fragile parts. Standalone or tethered VR kits generally have better audiovisual quality and interactivity but are often expensive, with tethered sets limited to locations with electrical outlets [25]. The MindCotine VR-MET environment included cue-exposure therapy using still images filmed with a 360 camera and animations for inducing meditative states. Interactivity beyond seeing and practicing cognitive behavioral strategies was not necessary for this intervention. It may be worthwhile to see the efficacy of CBT strategies before and after the intervention with interactivity in the VR environment (eg, users having the option of picking up a digital cigarette vs a digital substitute). However, this could also be done with smartphone app-based headsets where looking at an object long enough would count as a decision to use the object, as demonstrated in a smartphone app-based VR escape game [26].

Prior evaluations of VR interventions for smoking cessation largely studied adults outside of clinical settings and supported VR cue exposure therapy; more work is needed to explore multiple and combined treatment modalities and integration within clinical settings [18]. The study by Krebs et al [9], similar to our pilot in analytic sample size (n=8), but with a focus on patients recently hospitalized for cancer care, aimed to prevent relapse among those who recently quit smoking. Krebs et al [9] used a virtual world simulation game for treatment delivery to build skills to cope with smoking cues. Their game was found to cue cravings as designed, and the virtual practice of coping was viewed as helpful.

Our evaluation of the MindCotine VR intervention found that remote treatment and low-cost headsets for VR immersion are largely feasible and acceptable among patients receiving cancer care with varying levels of cigarette use. Quality testing onboarding procedures and adjustments with the headset are recommended prior to scaling to a remote study of feasibility, acceptability, and efficacy in a larger demographically diverse sample of patients undergoing cancer care.

### Conclusions

In this initial clinical pilot, the MindCotine VR intervention was of interest to more than 1 in 4 patients open to assistance with quitting smoking. Though remote onboarding and use of the technology proved challenging for some, most would recommend MindCotine to others, and a substantial proportion quit or reduced their smoking. The positive feedback and outcomes in reducing smoking from those who completed the intervention indicate that further testing of MindCotine in a larger sample is warranted. Further, uptake and sustained use are anticipated to be greater among people who are actively seeking strategies to quit smoking and perhaps among young adults and those familiar with digital games and VR environments.

## Abbreviations

CBT: cognitive behavioral therapy
HIPAA: Health Insurance Portability and Accountability Act
NRT: nicotine replacement therapy
REDCap: Research Electronic Data Capture
VR: virtual reality
VR-MET: virtual reality mindful exposure therapy

## Data Availability

The data sets generated or analyzed (or both) during this study are not publicly available due to restrictions for patient privacy protections.
